# RCSB Protein Data Bank: biological macromolecular structures enabling research and education in fundamental biology, biomedicine, biotechnology and energy

**DOI:** 10.1093/nar/gky1004

**Published:** 2018-10-24

**Authors:** Stephen K Burley, Helen M Berman, Charmi Bhikadiya, Chunxiao Bi, Li Chen, Luigi Di Costanzo, Cole Christie, Ken Dalenberg, Jose M Duarte, Shuchismita Dutta, Zukang Feng, Sutapa Ghosh, David S Goodsell, Rachel K Green, Vladimir Guranović, Dmytro Guzenko, Brian P Hudson, Tara Kalro, Yuhe Liang, Robert Lowe, Harry Namkoong, Ezra Peisach, Irina Periskova, Andreas Prlić, Chris Randle, Alexander Rose, Peter Rose, Raul Sala, Monica Sekharan, Chenghua Shao, Lihua Tan, Yi-Ping Tao, Yana Valasatava, Maria Voigt, John Westbrook, Jesse Woo, Huanwang Yang, Jasmine Young, Marina Zhuravleva, Christine Zardecki

**Affiliations:** 1 Research Collaboratory for Structural Bioinformatics Protein Data Bank, Rutgers, The State University of New Jersey, Piscataway, NJ 08854, USA; 2 Research Collaboratory for Structural Bioinformatics Protein Data Bank, San Diego Supercomputer Center, University of California, San Diego, La Jolla, CA 92093, USA; 3 Rutgers Cancer Institute of New Jersey, Rutgers, The State University of New Jersey, New Brunswick, NJ 08903, USA; 4 Institute for Quantitative Biomedicine, Rutgers, The State University of New Jersey, Piscataway, NJ 08854, USA; 5 The Scripps Research Institute, La Jolla, CA 92037, USA

## Abstract

The Research Collaboratory for Structural Bioinformatics Protein Data Bank (RCSB PDB, rcsb.org), the US data center for the global PDB archive, serves thousands of *Data Depositors* in the Americas and Oceania and makes 3D macromolecular structure data available at no charge and without usage restrictions to more than 1 million rcsb.org Users worldwide and 600 000 pdb101.rcsb.org education-focused Users around the globe. PDB Data Depositors include structural biologists using macromolecular crystallography, nuclear magnetic resonance spectroscopy and 3D electron microscopy. PDB *Data Consumers* include researchers, educators and students studying Fundamental Biology, Biomedicine, Biotechnology and Energy. Recent reorganization of RCSB PDB activities into four integrated, interdependent services is described in detail, together with tools and resources added over the past 2 years to RCSB PDB web portals in support of a ‘Structural View of Biology.’

## INTRODUCTION

The field of structural biology has been transformed by frequent advances in technology for every aspect of the structure determination pipeline since the Protein Data Bank (PDB) was established in 1971 (1) as the first open-access digital data resource in biology (2–6). Beginning with only seven protein structures, the PDB archive has ballooned to >145 000 structures of proteins, DNA, and RNA, and their complexes with metal ions and small molecule ligands (totaling >1 billion atoms).

Today, the PDB is universally regarded as a core data science resource of fundamental importance to the wider life-science community and long-term preservation of machine-readable biological data. PDB structures are the molecules of life. Knowledge of 3D structures (shapes) of biomolecules, how they evolve with time and how they function in nature is essential for understanding critical areas of science. PDB data impact basic and applied research on health and disease of humans, animals and plants; production of food and energy; and other research pertaining to global prosperity and environmental sustainability (7). Structure data are also important to biopharmaceutical and biotechnology companies, accelerating data-driven discovery of new drugs, materials and devices. Today, powerful pulsed X-ray facilities, cryogenic electron microscopes and new integrative/hybrid (I/H) methods for structure determination are accelerating biomedical research with functional insights into ever more complex biological systems at the atomic level. Cryo-electron tomography even allows study of molecular machines ‘caught in the act’ inside frozen cells.

Since 1999, Research Collaboratory for Structural Bioinformatics Protein Data Bank (RCSB PDB, rcsb.org) (3,7,8) has been funded by the NSF, NIH and DOE to safeguard and nurture the PDB archive and provide open access to PDB data. This enduring commitment reflects the critical importance of structure data to basic and applied research in Fundamental Biology, Biomedicine, Biotechnology and Energy. As a faithful steward of PDB data, RCSB PDB has transformed how the resource is managed as a global Public Good and how structure data are (i) expertly validated and biocurated when contributed by >30 000 PDB *Data Depositors*; (ii) stored in a relational database using an extensible common data standard; and (iii) packaged and delivered to >1 million PDB *Data Consumers*. Concurrently, RCSB PDB has kept pace with critical technical advances in macromolecular crystallography (MX) and nuclear magnetic spectroscopy (NMR) and the exciting developments of new structure determination methods [serial femtosecond X-ray crystallography and 3D electron microscopy (3DEM)], while engaging international experts and implementing community standards for data representation and validation.

PDB data address significant research questions in scientific disciplines ranging from Agriculture to Zoology (9,10). RCSB PDB delivers significant value to PDB *Data Consumers* providing important insights that go well beyond the content and scope of the original scientific publication. The RCSB PDB website (rcsb.org) provides researchers with a one-stop shop for 3D structure data. For each PDB structure, RCSB PDB integrates related data each week from ∼40 external resources and offers sequence and 3D structure visualization tools for researchers, educators and students. This unique combination of open access to primary and integrated data plus data analysis and structure visualization tools, enables 3D insights into molecular structure and function. RCSB PDB also provides tools for understanding collections of PDB structures, which in turn enables exploration of proteins from different organisms illuminating evolution at atomic and molecular levels. On its PDB-101 educational website (pdb101.rcsb.org), RCSB PDB provides introductory materials explaining fundamentals of protein, DNA and RNA structure; experimental methods used to generate PDB structures; and molecular stories highlighting Fundamental Biology, Biomedicine, Energy, Biotechnology and Drug Discovery. Compelling RCSB PDB usage and impact metrics underscore the importance of this resource to science and society, including >110 000 individual PDB structures contributing data to nearly 1 million scientific publications (as of February 2018); >1 million PDB Data Consumers served by rcsb.org in 2017; ∼680 million data files downloaded from the PDB archive in 2017; >620 000 PDB Data Consumers served by pdb101.rcsb.org in 2017; and PDB data reused by >400 external resources in 2017 (7,10).

In 2003, to ensure long-term sustainability of the PDB archive, RCSB PDB in the US worked with locally funded partners in Europe (Protein Data Bank in Europe, PDBe (11)) and Asia (Protein Data Bank Japan, PDBj (12)) to form the Worldwide Protein Data Bank (wwPDB, wwpdb.org) (2,5). wwPDB jointly manages the archive according to best practices, known as the ***FAIR*** principles (standing for ***F**indable*-***A****ccessible*-***I****nteroperable*-***R****eusable* (13)). The ***FAIR*** principles, developed by representatives from academia, industry, funding agencies and publishing, provide guidelines for data repositories to best support users and data reuse. Formation of the wwPDB has ensured that researchers, educators and students around the world enjoy open access to the world’s structure data following these guidelines. Formation of the wwPDB has also enabled equitable sharing of PDB data archiving and management costs between US, Europe, and Asia.

Since our last *Nucleic Acids Research* Database Issue publication (8), RCSB PDB activities have been reorganized into four integrated, interdependent cyberinfrastructures services, RCSB PDB hardware and software have been upgraded and new tools and resources have been introduced.

## REORGANIZATION OF RCSB PDB SERVICES

**Figure 1.**
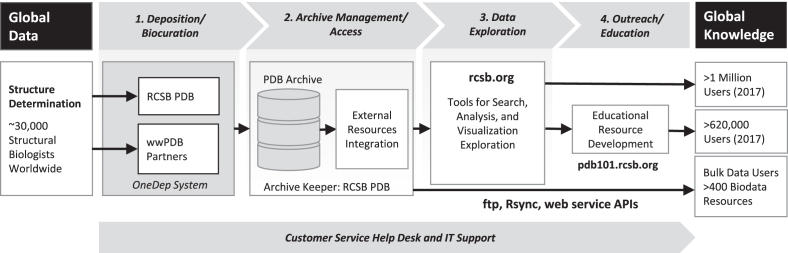
PDB data life-cycle and RCSB PDB services. RCSB PDB hosts four integrated, interdependent cyberinfrastructure services, supported by a Customer Service Help Desk and IT Support.

RCSB PDB activities were recently reorganized into four integrated, interdependent cyberinfrastructure services, including 1. *Deposition/Biocuration*; 2. *Archive Management/Access*; 3. *Data Exploration*; and 4. *Outreach/Education* (Figure 1). These new services were designed with the goal of improving the user experience and ensuring ongoing adherence to the ***FAIR*** principles (13).

### Deposition/Biocuration services ensure complete, *A*ccurate PDB data

RCSB PDB *Deposition/Biocuration* Services support *Data Depositors* in the Americas and Oceania, who contribute results of their structural studies of biomolecules to the PDB for archiving and data management (PDBe and PDBj support *Data Depositors* in Europe/Africa and Asia/Middle East, respectively.) PDB deposition is a prerequisite for publication of structural studies in most scientific journals and is typically required by public and private funders to ensure enduring public access to data. Key activities are as follows: (i) deposition-validation-biocuration support for submission of individual and groups of structures; and (ii) development of software supporting pre-deposition data preparation. Validation is critical for PDB *Data Consumers*, who rely on objective assessments of structure quality. Structure validation is also important for scientific publishing, and many journals require submission of PDB validation reports. Structure biocuration is critical for *Data Consumers*, who benefit from value-added information provided with each PDB structure.

Researchers around the world using two established methods (MX and NMR) and a third rapidly evolving method (3DEM) contribute data to the PDB archive via the wwPDB global deposition-validation-biocuration system, known as OneDep (14). PDB depositions include 3D structures (atomic coordinates), experimental data and metadata. OneDep is the product of an ongoing joint wwPDB development effort that began in 2008. Since 2014, OneDep has provided User Interfaces (UIs) for web-based deposition (14), validation (15), and biocuration (16) and a OneDep Workflow system that orchestrates and tracks tasks in the data pipeline.

In response to *Data Depositor* requests for parallel deposition of 10s–100s of related structures (typically the same protein with different bound ligands), RCSB PDB recently developed GroupDep (deposit-group.rcsb.rutgers.edu). Structures entering the PDB archive via GroupDep undergo validation-biocuration equivalent to those entering via OneDep. GroupDep was built atop RCSB PDB pre-deposition data capture/preparation software tools (17,18) that enable data file creation and consistency checking prior to submission.

Biocurators review and annotate each newly deposited structure to ensure accurate representation of both the structure and the underlying experimental data and related metadata. Using the OneDep system, the biocuration team reviews polymer sequences, small molecule chemistry, cross references to other databases, experimental details, correspondence of coordinates with primary data, protein conformation (Ramachandran plot), biological assemblies and crystal packing. Biocurators communicate with Depositors to ensure that the data are represented in the best way possible and are provided with good quality.

Once biocuration is complete, the final atomic coordinates, experimental data and metadata, and validation files, and a summary report, are made available at the OneDep Deposition User Interface, and the Data Depositor is invited by email to log back into the session and review the curated data files and the official wwPDB validation report. Following approval, the newly completed PDB entry is made public per release instructions and wwPDB policies (wwpdb.org/documentation/policy).

The Biocuration Team addresses questions submitted to the Customer Service Help Desk by Data Depositors and Data Consumers. Topics range broadly, and include questions about deposition process, data availability, system usability and more.

*Data Depositors* and Biocurators communicate via a secure, web-based interface integrated into the OneDep system, with email alerts for pending messages. Data Depositors provide corrections and annotations within the OneDep deposition interface.

### Archive Management/Access Services ensure *F*indable, *A*ccessible, *I*nteroperable and *R*eusable PDB data

RCSB PDB *Archive Management/Access* Services support *Data Consumers* worldwide. Key activities are as follows: (i) global archive keeping; (ii) data dictionary/data standardization; (iii) global data delivery and Digital Object Identifier (DOI) registration; and (iv) data integration. Related RCSB PDB software/data dictionaries are available in public code repositories (swtools.rcsb.org; mmcif.wwpdb.org; github.com/wwpdb-dictionaries).

Under the terms of the current wwPDB Agreement, RCSB PDB is the global *Archive Keeper*. RCSB PDB *Archive Management/Access* Services safeguard and maintain the PDB Core Archive, coordinating workflows globally for the weekly update and release of new and revised PDB data and the preservation of annual PDB archive snapshots. Multiple copies of the Core Archive are held in secure storage systems at both Rutgers and UCSD. In addition, RCSB PDB maintains redundant copies of a much larger collection of data files, documentation, and correspondence (∼50 TB) spanning the entire life of the PDB archive.

RCSB PDB *Archive Management/Access* Services ensure ***R****eusability* for *Data Consumers* by maintaining the PDB data dictionary and standard ontologies. Current RCSB PDB members led development of the PDBx macromolecular Crystallographic Information Framework (mmCIF) (19–23) as part of an International Union of Crystallography effort that began in the 1990s (24). In 2014, PDBx/mmCIF (mmcif.wwpdb.org) became the internationally recognized metadata standard for the PDB archive. RCSB PDB (with wwPDB partners and the wwPDB PDBx/mmCIF Working Group) coordinates PDBx/mmCIF development and hosts a public repository for data standards, metadata specifications, tutorials and links for accessing relevant software tools. The PDBx/mmCIF framework allows for automated checking of data consistency. PDB chemical and molecular data (25,26) are also managed with PDBx/mmCIF. As the archive grows and scientific sub-disciplines evolve, the way 3D structures are represented in the PDB requires ongoing adjustment (or ‘remediation’) to ensure consistency/accuracy. PDB data are regularly reviewed to identify data items that require improved representation to maintain the highest possible quality and utility of the archive (26–29).

RCSB PDB *Archive Management/Access* Services ensure ***F****indability* for *Data Consumers* by registering every PDB structure (currently >145 000) with a DOI. Access to individual structures and to specific data items for individual or multiple structures (e.g. bound ligand) is provided through RCSB PDB REpresentational State Transfer (or RESTful) web service Application Program Interfaces (APIs). Currently, these APIs support >80 selection queries that can recover all data pertaining to individual PDB structures or particular content details for individual or multiple structures. These same APIs are used by RCSB PDB *Data Exploration Services* described below.

In parallel, RCSB PDB *Archive Management/Access* Services ensure ***A****ccessibility* for *Data Consumers* to ∼1.4 million data files containing atomic coordinates, experimental data and related metadata (∼10 files/structure) with a total storage footprint of ∼1 TB. Versioned data are made available via file transfer protocol (ftp) and Remote sync (Rsync) download from Rutgers and UCSD, without access limitations or usage restrictions. N.B.: ftp and Rsync represent the means by which most biopharmaceutical and biotechnology companies access PDB data for proprietary research.

RCSB PDB *Archive Management/Access* Services support ***I****nteroperability* of PDB archive data with other biodata resources. For PDB *Data Consumers*, RCSB PDB integrates each PDB structure with data from ∼40 key resources by importing related information on a weekly basis (8). Highly time-intensive data integration functions, such as maintaining correspondence between the PDB archive and reference sequence databases, are managed collaboratively with wwPDB (e.g. SIFTS (30)). On the same weekly schedule, RCSB PDB pre-computes and stores comparative data derived from sequence and 3D structure similarity clustering to support PDB data ***F****indability* and ***I****nteroperability*.

### Data exploration Services ensure *F*indable, *A*ccessible and *R*eusable PDB data

RCSB PDB *Data Exploration* Services support PDB *Data Consumers* around the world through our open-access web portal (rcsb.org). Key activities are as follows: (i) hosting the rcsb.org website; (ii) providing services to find PDB structures; and (iii) providing services that enable understanding of PDB structures.

The RCSB PDB website provides facile online ***A****ccess* to every structure in the PDB archive with any of the popular browsers (e.g. Chrome, Firefox, Safari). Front-end software development uses Responsive Web Design technologies (31), supporting laptop/desktop computers, smart phones and tablet devices.

Within rcsb.org, an easy-to-use interface supports ***F****indability* with a system that searches for key data attributes and/or unstructured text. An autosuggestion function helps *Data Consumers* narrow search criteria efficiently. Search results can be viewed one structure at a time or summarized and sorted as tabular reports, which can be further refined or exported for ***R****euse*. Additional search options include taxonomy hierarchies, enzyme classifications, specific chemical components and similarity in sequence and/or 3D structure. Complex queries can be assembled by combining individual searches using our Advanced Search functionality.

RCSB PDB *Data Exploration* Services extend beyond simply delivering structure data, and well beyond what can be gleaned from the original scientific publication describing structure determination. Together, RCSB PDB *Archive Management/Access* and *Data Exploration* Services provide a one-stop shop for >1 million rcsb.org users annually, who want to understand any one of >145 000 PDB structures in the context of pre-organized scientific information drawn from ∼40 external biodata resources. The benefits are manifold. A one-stop-shop makes our *Data Consumers* more efficient users of structure data. Moreover, RCSB PDB provides them with access to a wide range of information that is updated weekly from resources that Users might not ordinarily consult. At last, sequence and 3D structure similarity data provided on rcsb.org help our Users make scientific connections that might otherwise have remained hidden (e.g. high-structure similarity of green fluorescent protein (PDB ID: 1ema (32)) and a mammalian basement membrane protein, Nidogen (PDB ID: 1gl4 (33)), despite low sequence identity ∼9%).

Once our Users have identified one or more structures of interest, RCSB PDB website features enable further exploration though mappings of structures to chromosomal positions and genetic variations (human only); metabolic pathways (human and *Escherichia coli*) (34); and information about drugs (DrugBank (35)) and ligands (BindingDB (36)). Sequence and 3D visualization tools include display of macromolecules and ligand interactions; electron density maps; structure validation information; and sites of post-translational and other chemical modifications (e.g. glycosylation) and biomedically important point mutations (37). Sequence/structure comparison tools provide insights into enzyme mechanism and selectivity, organization of macromolecular assemblies, evolutionary relationships among proteins and more.

### Outreach/Education Services support training and education via tailored *A*ccess to PDB data

RCSB PDB *Outreach/Education Services* are delivered via our PDB-101 website (pdb101.rcsb.org) (8), targeting PDB *Data Consumers* who may not be structural biologists or researchers (‘101’, denoting an entry level course). Simple text search tools support relatively inexperienced Users in ***A****ccessing* primary structural biology research data and learning about proteins, DNA, and RNA in 3D. The website ***I****nteroperates* seamlessly with the PDB archive and rcsb.org. As PDB-101 Users gain more experience with PDB data, they naturally begin using rcsb.org, wherein RCSB PDB *Data Exploration* Services reveal the fullness of the PDB archive. Many of our experienced *Data Consumers* report frequenting both rcsb.org and pdb101.rcsb.org websites, attesting to the enduring value of the introductory and training materials provided by our public outreach activities. PDB-101 was highlighted as ‘Best of the Web’ by *Genetic Engineering & Biotechnology News* in 2017 (38).

For educators, students, and the public, PDB-101 also develops resources that use PDB structures to tell the molecular stories surrounding a biennial Health Focus. For the 2018–2019 topic of Antibiotic Resistance, PDB-101 hosts a video challenge for high schools, publishes new articles and features, and develops curricular modules.

### RCSB PDB hardware and software architecture upgrades

**Figure 2.**
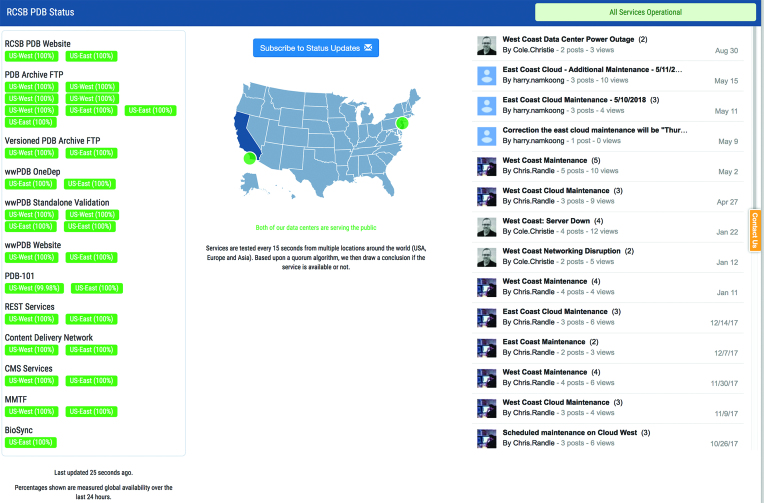
RCSB PDB Services Status page. Public accessibility of all critical project services is monitored and displayed on a dedicated webpage (status.rcsb.org). Percentages shown reflect the availability level of the resource over the previous 24-h period. Service interruptions trigger automatic redirection of User traffic between Rutgers and UCSD, and staff notifications to ensure prompt evaluation resolution. From this page, users can subscribe to an electronic list for related notifications (Status Updates).

The four RCSB PDB services are deployed on advanced cyberinfrastructure that is scalable to meet variable demand providing >99% uptime 24 × 7 × 365 (housed at both Rutgers and UCSD). All critical project services are monitored by our commercial Domain Name System provider (ns1.com) and publicly displayed on a dedicated webpage (status.rcsb.org, Figure 2). Service interruptions trigger automatic redirection of User traffic between Rutgers and UCSD, and staff notifications to ensure prompt evaluation and resolution. Bi-coastal deployment has allowed scaling of ftp, Rsync and RESTful web services to meet our Service Level Objective of >99% uptime 24 × 7 × 365.

Three of the four RCSB PDB services (*Archive Management/Access, Data Exploration* and *Outreach/Education Services*) are deployed on a bicoastal private cloud based on open-source software (e.g. OpenStack Nova, Cinder). Multiple copies (or instances) of these services are deployed on both coasts for load balancing and failover. During calendar year 2017, *Data Exploration* services on rcsb.org were accessed by ∼9.7M unique visitors (IP addresses) with an associated bandwidth load of 37 TB/year.

## NEW RCSB PDB TOOLS, DATA AND RESOURCES

### RCSB PDB microservices

Recent cyberinfrastructure improvements described above provide faster access to rcsb.org content with improved page load times. RCSB PDB is currently moving to a new microservice-based architecture to better scale our service demands to accommodate growth of the PDB archive and increased *Data Consumer* demands, and increase the speed at which we can deploy new services. In parallel, URLs are being streamlined for easier access and sharing. For example, the Structure Summary page previously at the URL https://www.rcsb.org/pdb/explore/explore.do?structureId=4dkl is now accessed using https://www.rcsb.org/structure/4dkl.

Structure Summary pages on rcsb.org that utilize the new microservice architecture have enabled faster access to macromolecule sequence information, biological assembly evidence for recent structures, software packages used, deposition identifiers for large groups of related structures submitted and more.

REST microservices used internally to support new features at rcsb.org are also available for public use (rest.rcsb.org).

### Integration with external data resources

As part of the weekly update of carried out within the RCSB PDB *Archive Management/Access* services, PDB structure data are integrated with corresponding information from ∼40 external data resources (Table 1). These data are then made accessible from Structure Summary pages and rcsb.org searching and reporting tools. Representative examples include diffraction image data and structural flexibility data.

**Table 1.** External data resources integrated with PDB data

External Resource	URL	Type of Data
BiGG	bigg.ucsd.edu	Reconstruction of metabolic pathways
Binding MOAD	bindingmoad.org	Binding affinities
BindingDB	bindingdb.org	Binding affinities
BMRB	www.bmrb.wisc.edu	BMRB-to-PDB mappings
Catalytic Site Atlas	www.ebi.ac.uk/thornton-srv/databases/CSA	Active sites and catalytic residues in enzymes
CATH	www.cathdb.info	Protein structure classification
DrugBank	www.drugbank.ca	Drug and drug target data
EMDB	pdbe.org/emdb/	3DEM density maps and associated metadata
ExPASy	expasy.org	Enzyme classification
Gencode	www.gencodegenes.org	Gene structure data
Gene Ontology	www.geneontology.org	Biological ontologies
HMMER3	hmmer.janelia.org	Sequence similarity searches
Human Gene Nomenclature Committee	www.genenames.org	Human gene name nomenclature and genomic information
Immune Epitope Database	www.iedb.org	Antibody and T-cell epitopes
LS-SNP	ls-snp.icm.jhu.edu/ls-snp-pdb	Single Nucleotide Polymorphisms
Mpstruc	blanco.biomol.uci.edu/mpstruc	Classification of transmembrane protein structures in PDB
NCBI Gene	www.ncbi.nlm.nih.gov/gene	Gene info, reference sequences, *et al.*
NCBI Taxonomy	www.ncbi.nlm.nih.gov/taxonomy	Organism classification
NDB	ndbserver.rutgers.edu	Experimentally determined nucleic acids and complex assemblies
OLDERADO	www.ebi.ac.uk/pdbe-apps/nmr/olderado	NMR domain composition and clustering
OPM	opm.phar.umich.edu	Orientation of transmembrane proteins
PDBbind-CN	www.pdbbind-cn.org	Binding affinities
PDBflex	pdbflex.org	Protein structure flexibility
Pfam	pfam.sanger.ac.uk	Protein families
PhospoSitePlus	www.phosphosite.org	Mammalian post-translational modifications
Protein Model Portal	www.proteinmodelportal.org	Homology models
ProteinDiffraction.org	proteindiffraction.org	Diffraction images
PubMed	www.ncbi.nlm.nih.gov/pubmed	Citation information
PubMedCentral	www.ncbi.nlm.nih.gov/pmc	Open access literature
RECOORD	www.ebi.ac.uk/pdbe/recalculated-nmr-data	NMR structure ensembles
RESID	pir.georgetown.edu/resid	Protein modifications
SBGrid	sbgrid.org	Structural Biology Data Grid/diffraction images
SCOP	scop.mrc-lmb.cam.ac.uk/scop	Protein structure classification
SIFTS	www.ebi.ac.uk/pdbe/docs/sifts	Structure, function, taxonomy, sequence
Store.Synchrotron Data Store	store.synchrotron.org.au	Diffraction images
Transporter Classification Database	www.tcdb.org	Classification of membrane transport proteins
UCSC genome browser	genome.ucsc.edu	Human genome data
UniProt	www.uniprot.org	Protein sequences and annotations

This list is maintained at www.rcsb.org/pages/external-resources.

Several resources that store diffraction image data related to PDB structures have been established recently. Such data are made available to help improve the reproducibility of structural biology studies and the automation of structure determination tools. RCSB PDB now links to diffraction image data from the Store.Synchrotron Data Store (Store.Synchrotron.org.au) in addition to the Structural Biology Data Grid (sbgrid.org) and proteindiffraction.org.

Proteins frequently display evidence of conformational flexibility, when different PDB structures of the same protein are compared. In many cases, this deformability is functionally relevant. Information regarding structural variation represented in similar amino acid sequences has been available through the integration of PDB structures with data from the PDBFlex database (39). The PDBFlex database explores the intrinsic flexibility of protein structures by analyzing structural variations of the same protein across the archive. Such comparisons allow for the easy identification of regions and types of structural flexibility present in a protein of interest. Structures of polypeptide chains with nearly identical sequences (sequence identity > 95%) are aligned, superimposed and clustered. Identification of similar sequences in this report is based on the clustering used by RCSB PDB.

### Improved text searching

With access to newer technologies, simple text searches at rcsb.org have been considerably improved, enabling easier and more accurate interrogation of PDB data. Text searching from the top query bar combines the power of the open source Apache Solr platform and full indexing of PDBx/mmCIF data.

**Figure 3.**
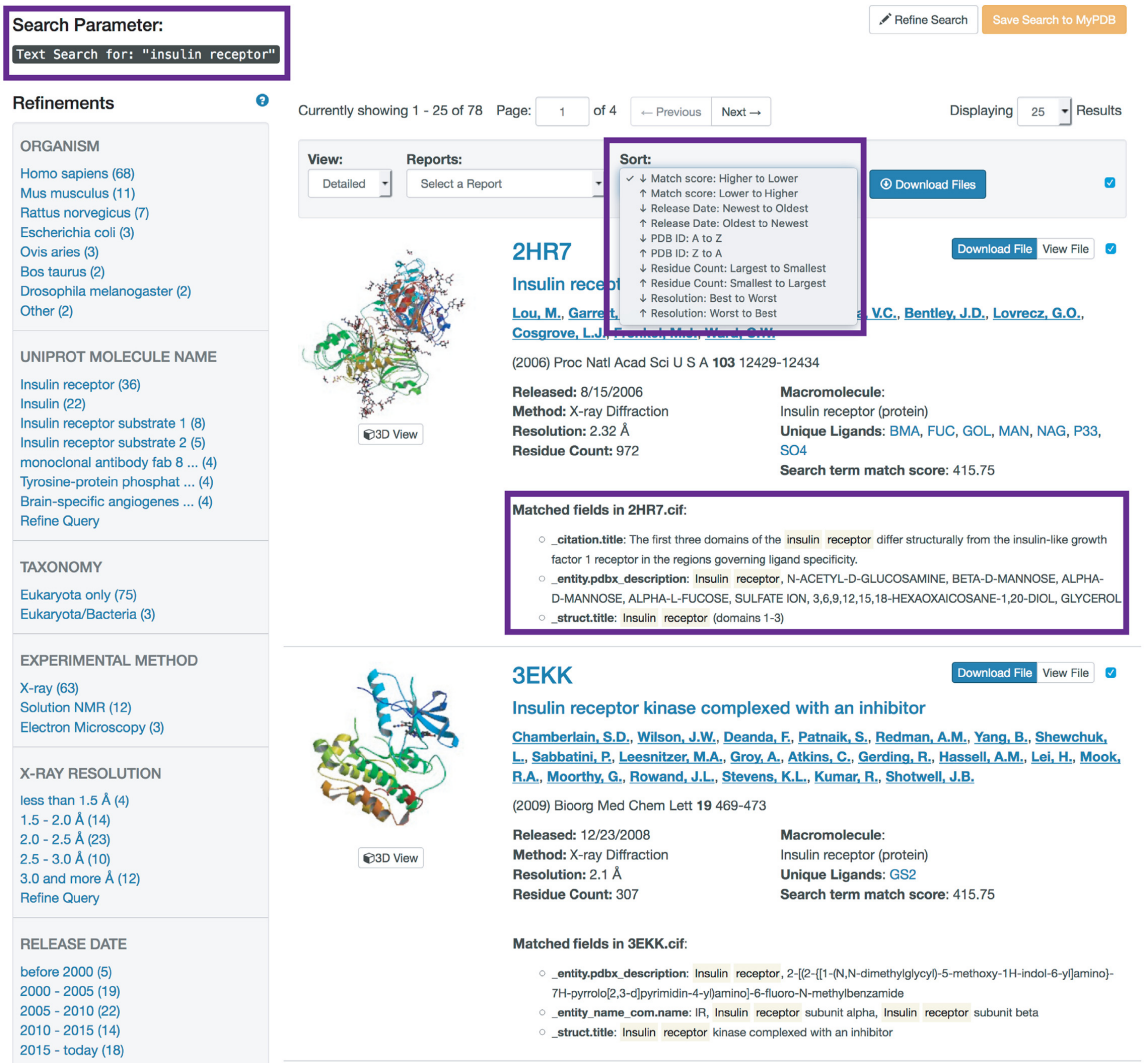
Text search results and options for exploration. Search terms ‘insulin receptor’ enclosed in double quotation marks are indicated on upper left of page. For each entry in the search results, the appearance of the search term in categories relating to author, citation, entity name, entity description, keyword or title is highlighted (under ‘Matched fields,’ highlighted in lower right box). Search results can be sorted by match score, release date, PDB ID, residue count or resolution (highlighted in upper right box). Custom and default reports can be generated and downloaded. Users can also access corresponding data files and Structure Summary pages.

Users may access this new functionality by entering a search term or terms in the top bar of any RCSB PDB webpage and clicking the ‘Go’ button or issuing a keyboard return (Figure 3). Searches for multiple words (for example, insulin receptor) and queries for adjacent words enclosed in double quotation marks (for example, ‘insulin receptor’) are intended to return different results. The first search finds results wherein the words appear anywhere in the entry, whereas the second returns results wherein the search terms appear exactly as ordered.

Search results are assigned ‘Match Scores’ to help indicate the relevance of the result and to sort structures from ‘Higher to Lower’ matches and *vice versa*. Search results can also be sorted according to ‘Release Date’ Oldest to Newest or Newest to Oldest; ‘PDB ID’ A to Z or Z to A; ‘Residue Count’ Largest to Smallest or Smallest to Largest; and ‘Resolution’ Lowest to Highest or Highest to Lowest.

### Rapid visualization of complex PDB structure data

**Figure 4.**
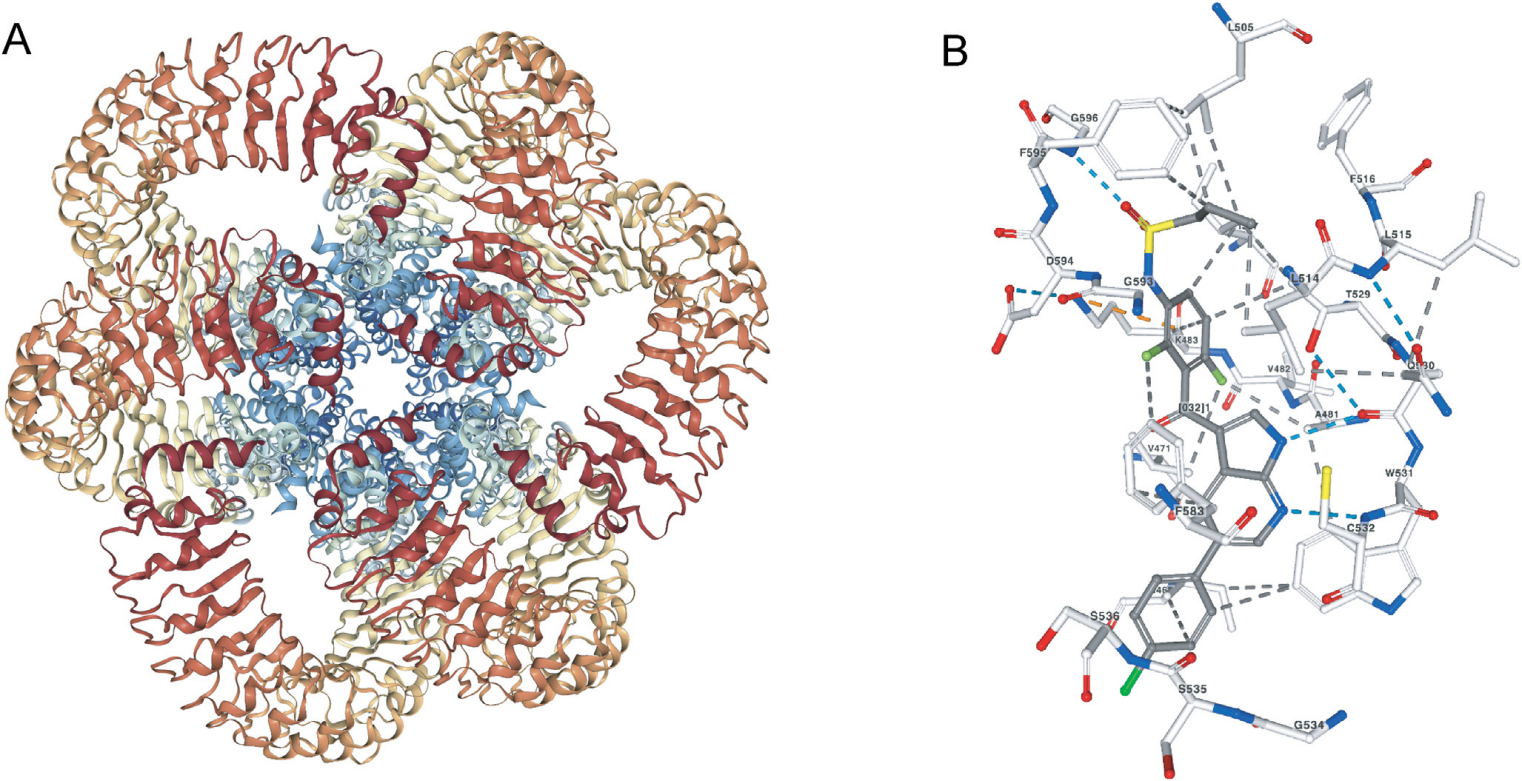
Features of the NGL 3D Viewer. (**A**) NGL view of the structure of a hexameric, volume-regulated anion channel of the LRRC8 family (PDB ID: 6g9l (54)), viewed down the ion conducting pore from the cell surface. Polypeptide chain ribbons are colored from N-terminus (blue) to C-terminus (red). Polypeptide chain ribbons are colored from N-terminus (blue) to C-terminus (red). (**B**) NGL view of the interaction of B-Raf Kinase bound to the US FDA approved anti-neoplastic drug Vemurafenib (PDB ID: 3og7 (55)). Ball-and-stick figure atom color coding (C-gray for drug or white for protein; O-red; N-blue; S-yellow; F-green). Hydrogen bonds are denoted with blue dashed lines, hydrophobic interactions with gray-dashed lines and cation–π interactions with pink-dashed lines.

RCSB PDB Structure Summary pages on rcsb.org also offer fast, interactive 3D display of molecular complexes containing millions of atoms on desktop computers (without any special plug-ins) and even smartphones and tablets using the NGL Viewer [Figure 4, (40,41)]. NGL Viewer uses an internally developed binary compressed format (Macromolecular Transmission Format) that considerably reduces network transfer and parsing time requirements (42).

The NGL Viewer offers three main views to access Structure, Electron Density, and Ligands in 3D. In addition to the standard features offered for full Structure viewing (e.g., color, representation style), new options in the NGL viewer map wwPDB Validation Report information onto the 3D structure. These same wwPDB Validation Reports are publicly available, helping to identify structures of sufficient quality and accuracy for intended study. They are also intended to help ensure the integrity of the peer-reviewed scientific literature. Access to validation reports helps referees and editors better evaluate the structure and improve publication quality. NGL can be used to highlight interatomic clashes and to display the full structure using ‘Geometry Quality’ and ‘Density Fit’ coloring schemes.

To explore macromolecular-ligand interactions, Ligand Interaction viewing (Figure 4B) features include options to display the surface of the ligand binding pocket and non-covalent interactions (hydrophobic contacts, hydrogen bonds, halogen bonds, metal interactions, π–π interactions) between the ligand and the macromolecule. Calculations are performed in real-time within the web browser. This easy-to-use feature is particularly important for the majority of rcsb.org users, who are not structural biologists. Facile display and interrogation of ligand binding properties enable design of hypothesis testing studies by molecular biologists (e.g. site-directed mutagenesis of amino acid involved in ligand binding) and support structure-based drug design.

NGL also displays experimental data coming from MX in the form of electron density maps. Both 2|Fobserved|-|Fcalculated| (blue mesh/surface) and |Fobserved|-|Fcalculated| (red/green mesh/surface) difference maps can be displayed together with the atomic structure of the macromolecule. Facile review of these electron density maps is essential for interpreting MX structure data. For example, rcsb.org Users can now judge for themselves whether or not the fit of an ostensibly bound ligand in the electron density supports earlier claims made by the structural biologist(s) that originally published the structure. Moreover, rapid access to electron density maps can also reveal regions of structures that were not well-resolved in the MX experiment, providing the impetus for complementary biological and functional studies.

### Hosting the EPPIC resource

**Figure 5.**
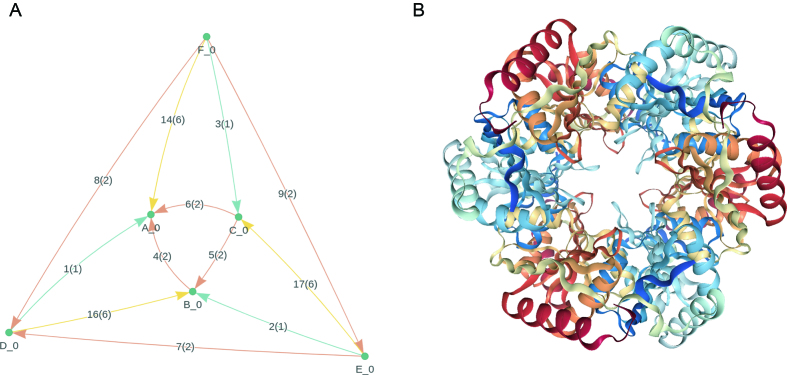
EPPIC and 3D NGL ribbon diagram views of CcmP, a tandem bacterial microcompartment domain protein from the beta-carboxysome (PDB ID: 4ht5 (56)). (**A**) EPPIC assembly graph corresponding to the D3 symmetric assembly with the NGL view of the same assembly. Nodes denote proteins and edges denote interfaces between proteins, colored to represent distinct modes of protein–protein interaction. (**B**) Same structure in NGL viewer. Polypeptide chain ribbons are colored from N-terminus (blue) to C-terminus (red).

EPPIC (Evolutionary Protein–Protein Interface Classifier) provides value-added information about biological assemblies in the PDB (43). This web server classifies interfaces present in protein crystals to distinguish biological interfaces from crystal contacts (Figure 5). The latest version of EPPIC (v3) enumerates all possible symmetric assemblies with a prediction of the most likely assembly based on probabilistic scores from pairwise evolutionary scoring. EPPIC is now fully hosted and supported by RCSB PDB at eppic-web.org.

### PDB archive metrics

**Figure 6.**
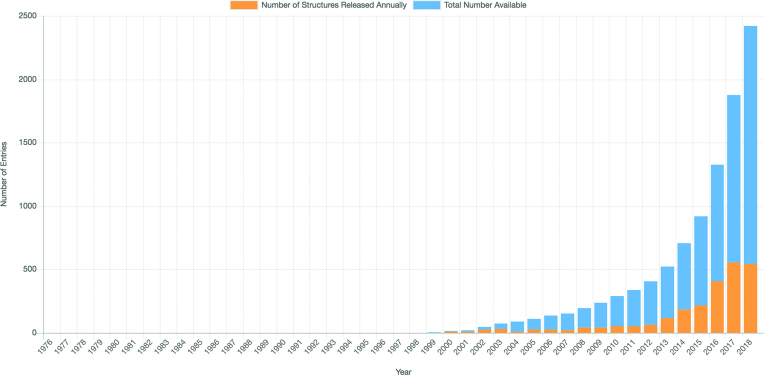
PDB Metrics: growth of new 3DEM structures in the PDB. All statistical charts are updated dynamically each week. Data can be downloaded for external use. This 3DEM growth chart can be accessed at www.rcsb.org/stats/growth/em.

Improved displays of PDB metrics have recently been made available. These PDB statistics are generated using RESTful services to dynamically represent the current holdings of the archive. Examples include distribution of data by experimental method, enzyme classification, organism and journal. Growth charts track the number of structures released per year by experimental method and macromolecular structure classification. The corresponding tabular data can be downloaded. For example, Figure 6 illustrates the very rapid growth in the number of 3DEM structures released annually that has occurred since 2012, highlighting the impact of a new generation of cryogenic transmission electron microscopes and direct electron detectors.

## SUMMARY

RCSB PDB has evolved considerably since its first *NAR Database Issue* publication nearly two decades ago (3), driven by the needs of a growing and diverse User community that now exceeds 1 million individuals worldwide. In 2014, the inaugural RCSB PDB Berman *et al.* (3) article was ranked 92nd in the top 100 all time cited publications by the Web of Science (44), thus providing a useful data set for bibliometric analyses. A 2017 study of this publication performed for RCSB PDB by Clarivate Analytics documented that the PDB motivated high-quality research around the world (9). The Citation-based Impact of publications citing Berman *et al.* (3) exceeded the world-average in a total of 16 distinct scientific fields, including Biology & Biochemistry, Computer Science, Plant & Animal Sciences, Physics, Environment/Ecology, Mathematics and Geosciences. A complementary bibliometric study of the impact of the RCSB PDB revealed that the annual number of citations of Berman *et al.* (3) has been consistently high, with an average of ∼940 citations/year since 2004 (10). Additional *NAR* Database Issue articles provide valuable updates on the development of RCSB.org, and are also well-cited (8,45–53).

In 2021, the Protein Data Bank will celebrate its 50th year of operations. Reorganization of the resource around four integrated, interdependent cyber infrastructure services and strengthening of the hardware and software architecture through the use cloud computing and microservices will position the RCSB PDB to continue supporting the community for the next 50 years.

## DATA AVAILABILITY

RCSB PDB services are available from http://rcsb.org.
